# A robust framework to investigate the reliability and stability of explainable artificial intelligence markers of Mild Cognitive Impairment and Alzheimer’s Disease

**DOI:** 10.1186/s40708-022-00165-5

**Published:** 2022-07-26

**Authors:** Angela Lombardi, Domenico Diacono, Nicola Amoroso, Przemysław Biecek, Alfonso Monaco, Loredana Bellantuono, Ester Pantaleo, Giancarlo Logroscino, Roberto De Blasi, Sabina Tangaro, Roberto Bellotti

**Affiliations:** 1 Dipartimento di Fisica, Università degli Studi di Bari Aldo Moro, Bari, Italy; 2 Istituto Nazionale di Fisica Nucleare, Sezione di Bari, Bari, Italy; 3 Dipartimento di Farmacia - Scienze del Farmaco, Università degli Studi di Bari Aldo Moro, Bari, Italy; 4 Faculty of Mathematics and Information Science, Warsaw University of Technology, Warsaw, Poland; 5 Faculty of Mathematics, Informatics and Mechanics, University of Warsaw, Warsaw, Poland; 6 Dipartimento di Scienze mediche di base, Neuroscienze e Organi di senso, Università degli Studi di Bari Aldo Moro, Bari, Italy; 7 Pia Fondazione “Card. G. Panico”, Tricase, Italy; 8 Dipartimento di Scienze del Suolo, della Pianta e degli Alimenti, Università degli Studi di Bari Aldo Moro, Bari, Italy

**Keywords:** Alzheimer’s disease, Cognitive spectrum, Explainable Artificial Intelligence, Mild Cognitive Impairment, XAI

## Abstract

In clinical practice, several standardized neuropsychological tests have been designed to assess and monitor the neurocognitive status of patients with neurodegenerative diseases such as Alzheimer’s disease. Important research efforts have been devoted so far to the development of multivariate machine learning models that combine the different test indexes to predict the diagnosis and prognosis of cognitive decline with remarkable results. However, less attention has been devoted to the explainability of these models. In this work, we present a robust framework to (i) perform a threefold classification between healthy control subjects, individuals with cognitive impairment, and subjects with dementia using different cognitive indexes and (ii) analyze the variability of the explainability SHAP values associated with the decisions taken by the predictive models. We demonstrate that the SHAP values can accurately characterize how each index affects a patient’s cognitive status. Furthermore, we show that a longitudinal analysis of SHAP values can provide effective information on Alzheimer’s disease progression.

**Supplementary Information:**

The online version contains supplementary material available at 10.1186/s40708-022-00165-5.

## Introduction

Alzheimer’s disease (AD) is a neurodegenerative pathology caused by multiple factors whereby neurological changes in the brain could occur several decades before cognitive impairment arises [1]. Typically, a long time may elapse between the onset of the disease (characterized by memory problems and difficulties in learning new information) and the full manifestation of symptoms of dementia in which patients become unable to complete basic daily life activities [2]. The progression of the disease is naturally described as a continuous spectrum ranging from preclinical and prodromal stages to the dementia syndrome onset [3, 4]. Nevertheless, the high variability affecting this spectrum during aging in both performance levels [5] and cognitive functions [6], makes the use of broad categories, such as cognitively normal, mild cognitive impairment (MCI) and dementia, a convenient representation [7]. In particular, MCI is considered a transitional stage between healthy aging and dementia. MCI patients have complaints of cognitive deficits that do not interfere significantly with daily life activities [8, 9]. However, using a single diagnostic category for a condition that can affect heterogeneous domains (e.g., memory, attention, executive functions, language and visuospatial abilities [10]) to very different extents is controversial [11–14]. In this context, testing cognition in a large elderly population regularly, before major memory loss, might help to capture and understand the cognitive variability between normal and MCI conditions at risk of later developing dementia. Thus, a deeper understanding of the mechanisms of this variability could help physicians make decisions about treatment options or patients’ enrollment in cognitive rehabilitation programs as well as clinical trials for novel drugs [15, 16].

Several tests provide information about the neuropsychological conditions of patients and are commonly adopted to assess the severity of the most important symptoms of AD [17]. The most commonly used cognitive indexes include: the Alzheimer’s Disease Assessment Scale cognitive total score (ADAS), Mini Mental State Exam score (MMSE) and the Rey Auditory Verbal Learning Test (RAVLT) which measures cognitive impairment, attention, language and visuospatial functions, and memory deficits. Different scores resulting from neuropsychological evaluations have been widely used to model disease progression [18, 19], for early diagnosis of AD [20] and to detect subtypes of dementia [21]. In particular, in the last 2 decades, there has been an exponential growth in machine learning (ML) applications for both the prognosis and diagnosis of dementia. Both supervised and unsupervised methods have been effectively used to perform multiclass classification of different diagnostic categories [22, 23], to predict the risk of conversion from mild cognitive impairment to Alzheimer’s disease [24, 25] and to categorize and cluster the clinical data [26, 27].

Although ML models for the diagnosis and prognosis of cognitive decline and Alzheimer’s disease have reached very high-performance levels, most works have devoted little attention to the explainability of these models. Indeed, although these models achieved tremendous predictive performance, they are not gaining popularity in clinical practice due to their high inner complexity. Explainability is the ability to explain AI decision-making in human-understandable ways to a wider variety of end-users [28]. Of course, different end-users are interested in different aspects of explainability. For example, data scientists and developers could be more interested in the explainability of the algorithms, while medical professionals and physicians could be primarily concerned with clinical prediction. More recently, eXplainable Artificial Intelligence (XAI) methods have been developed to estimate the contribution of individual features towards specific predictions, thus generating a set of feature importances for each patient [29]. The field of eXplainable Artificial Intelligence has rapidly advanced in recent years as ML models are becoming more and more popular in different application areas. As a result, a huge and increasing number of issues are being addressed, including the negative aspects of automated applications such as possible biases and failures, which in turn have led to the development of new ethical guidelines and regulations [30, 31]. In April 2019, the European Commission High-Level Expert Group on AI presented “Ethics Guidelines for Trustworthy Artificial Intelligence” and three of the guidelines directly refer to explainability [31]. Several criteria exist to classify XAI methods. Although a unified taxonomy has not been established, basically, XAI methods can be grouped into intrinsic or post-hoc [32]. The first group includes machine learning techniques that are interpretable due to their internal structure, such as linear models and decision trees, while the second category includes methods for analyzing the ML models after they have been trained [33]. Different local post-hoc XAI algorithms have been developed to provide single instance explanations regardless of the specific predictive model, making these techniques fully adaptable to the clinical context and personalised medicine, including individualised interventions and targeted treatments [34–36].

While these XAI approaches have proved useful in certain medical AI fields, notably in visualization, virtual reality and other Big data problems [37, 38], their application to the domains of cognitive and computational neuroscience is still in its infancy. In addition, no unified quantitative metrics have been defined to evaluate the reliability of the methods, raising questions about the consistency of numerical explanations depending on the specific applications. For example, in clinical classification tasks, several issues may arise when using XAI methods: (1) to what extent are explainability values related to the efficiency of the ML algorithms? (2) Do groups of subjects with similar characteristics actually exhibit similar explainability scores? (3) Can XAI values in turn be used to provide insights into the state of disease progression?

In this paper, we present a framework based on ML, XAI and statistical analysis to perform a three-classes classification between healthy control subjects, individuals with different levels of cognitive impairment, and subjects with dementia by using different cognitive indexes and to analyze the variability of the explainability values associated with the decisions taken by the predictive models. The present work aims to: (i) relate the performance of the ML algorithm to the variability of the extracted XAI values by applying a local post-hoc algorithm; (ii) analyze any potential differences between the explainability values associated with both correctly and incorrectly classified subjects; (iii) identify subgroups of subjects by applying unsupervised algorithms to their explainability values in order to provide the physicians with a set of predictors most associated with the subgroups; (iv) examine the longitudinal variations of the XAI values to identify the cognitive indexes whose importance is most variable in subjects converting from one diagnostic category to another and provide the clinicians with a list of cognitive indexes best suited to track cognitive variations across the spectrum of neurodegeneration.

We hypothesise that the explainability indexes could themselves be considered markers to describe both the change in cognitive status of patients during nominal and pathological ageing and to reliably quantify the contribution of each functional cognitive domain to the overall condition of each patient. Thus, XAI scores could provide personalized neurodegeneration patterns, explaining the heterogeneous contributions of the functional domains to AD. This latter aspect is particularly crucial to identifying an optimal set of predictors to classify AD.

## Related works

So far, XAI methods have been applied to computational neuroscience in a few cases to explore how different brain regions affect age prediction or to predict the onset of cognitive decline. These aspects are particularly relevant given the risk that cognitive impairment could convert to AD.

Specifically, Beebe-Wang et al. [39] developed a machine learning model and tested it in an aging cohort study with an extensive set of longitudinal clinical variables to highlight at-risk individuals with better accuracy than other approaches. They selected a subset of highly predictive cognitive tests and used XAI scores to provide individualized prediction explanations that retain non-linear feature effects present in the data.

El-Sappagh et al. [40] developed a ML model for AD diagnosis and progression detection by integrating 11 modalities of 1048 subjects from the Alzheimer’s Disease Neuroimaging Initiative (ADNI) dataset. The model was designed as a two-layer framework with a random forest (RF) classifier algorithm. In the first layer, the model carries out a multi-class classification for the early diagnosis of AD patients. In the second layer, the model applies binary classification to detect possible MCI-to-AD progression within three years from the baseline diagnosis. Moreover, for each layer, instance-based explanations of the RF classifier were provided by the SHapley Additive exPlanations (SHAP) to indicate the feature importance.

In a previous work [41], we exploited an explainable Deep Learning-based framework to both maximize the brain age prediction accuracy and achieve high interpretability of the contribution of each brain morphological feature extracted from magnetic resonance imaging (MRI) of a healthy cohort of subjects to the final predicted age by exploiting local XAI algorithms.

In addition, different visual XAI techniques have been developed to quantify the interpretability of the latent representations of CNNs for the classification of dementia and cognitive impairment, such as the layer-wise Relevance Propagation (LRP) technique [42], saliency maps, and Gradient-weighted Class Activation Mapping (Grad-CAM) [43]. Such methods can be used to produce coarse localization maps, highlighting the important regions in each MRI scan by exploiting voxel-level information. More recently, Generative Adversarial Network (GAN)-based methods are gaining popularity for understanding the areas of T1 scans that affect the classification process of AD and MCI. Yu et al. [44] proposed a tensorizing GAN with High-order pooling to assess MCI and AD in a semi-supervised manner taking advantage of both labelled and unlabeled MRI scans while automatically extracting significant features in a self-attention manner. The authors also implemented a multidirectional mapping mechanism for a novel GAN architecture to provide class discriminative maps of the whole brain [45].

## Materials

### Study cohort

In this study, we exploited a dataset obtained from the Alzheimer’s Disease Neuroimaging Initiative (ADNI) database (http://adni.loni.usc.edu/). ADNI researchers collected several types of data from study volunteers throughout their participation in the study. Data collection was performed using a standard set of protocols and procedures to eliminate inconsistencies. Subjects have been recruited from over 57 sites across the US and Canada. The study was conducted according to the Good Clinical Practice guidelines, the Declaration of Helsinki, and US 21 CFR Part 50 (Protection of Human Subjects), and Part 56 (Institutional Review Boards). Subjects were willing and able to undergo test procedures, including neuroimaging and follow-up, and written informed consent was obtained from participants.

We used the ADNIMERGE R package to download the data on June 1, 2021 (see www.adni-info.org). Up to the date of June 1, 2021, 15,304 samples belonging to 2306 adults, ranging in age from 55 to 90, have been collected. In this study, we employed data collected at baseline visits and longitudinal data at successive visits. Initial preprocessing of the data revealed that the pattern of data missingness was correlated with the diagnosis, as, for example, some measurements were missing more often in healthy subjects. We, therefore, kept clinical and neuropsychological variables and samples with no missing entries. This step led to a reduction in the number of variables to 10 and the number of samples from 15,304 to 6285.

The 6285 samples (53.3% male) were categorized into three groups according to the diagnosis given at the exam date: (a) normal controls (NC): 2408 samples (38.3%), (b) MCI: 2912 samples diagnosed as MCI (46.3%), (c) AD: 965 samples diagnosed with dementia (15.4%). Demographic, clinical and neuropsychological information of the samples is reported in Table 1. All the analyses were performed using R (version 3.6.2) software.

**Table 1:** Demographic, clinical and neuropsychological information on the selected cohort

	NC	MCI	AD
Age (years)	$75.2 \pm 7.1$	$74.5 \pm 7.9$	$76.5 \pm 7.5$
Education (years)	$16.6 \pm 2.4$	$16.1 \pm 2.7$	$15.9 \pm 2.5$
Gender (M/F)	1082/1326	1691/1221	577/388
ADAS11	$6.7 \pm 3.2$	$9.6 \pm 4.7$	$20.6 \pm 7.3$
ADAS13	$10 \pm 4.7$	$15.1 \pm 7.1$	$30.9 \pm 9$
MMSE	$29 \pm 1.2$	$27.8 \pm 1.9$	$22.5 \pm 3.6$
RAVLT immediate	$46.5 \pm 10.8$	$36.6 \pm 11.2$	$22.5 \pm 7.9$
RAVLT learning	$5.9 \pm 2.4$	$4.5 \pm 2.6$	$1.9 \pm 2.8$
RAVLT percforgetting	$34.2 \pm 29.7$	$56.7 \pm 47.2$	$90.2 \pm 25.6$
FAQ	$0.2 \pm 0.9$	$2.7 \pm 3.9$	$15.4 \pm 7.2$
MOCA	$25.9 \pm 2.5$	$23.5 \pm 3.2$	$17.3 \pm 4.7$
EcogPtTotal	$1.4 \pm 0.3$	$1.7 \pm 0.5$	$1.9 \pm 0.6$
EcogSPTotal	$1.2 \pm 0.2$	$1.7 \pm 0.6$	$2.9 \pm 0.6$

### Clinical and neuropsychological assessment

The ADNI database contains more than 40 variables resulting from different cognitive and functional assessments. In this work, the 10 indexes: ADAS11, ADAS13, MMSE, MoCA, FAQ, RAVLT-immediate, RAVLT-learning, RAVLT-percforgetting, ECogPt total and ECogSP total were selected to reflect the condition and functionality of each subject at each visit. The selected indexes encompass scores of different neuropsychological tests’ ratings and standard questionnaires both of participant or study partner (SP), and are widely adopted as screening tools to detect memory deficits and behavioral symptoms associated with dementia. The indexes are briefly described in Table 2. More details about the assessments and procedures administered to the subjects can be found on the web page of the Procedures Manual of the study (http://adni.loni.usc.edu/wp-content/uploads/2012/10/ADNI3-Procedures-Manual_v3.0_20170627.pdf).

**Table 2:** Description of the selected clinical and neuropsychological indexes

Index	Description
ADAS11	A test that is composed of 11 tasks to assess cognitive functioning of memory, praxis and language. Specific tasks include Naming Objects, Word Recall, Fingers, Commands, Orientation, Word Recognition, Constructional Praxis, Ideational Praxis and Language. [46]
ADAS13	A test including all elements of ADAS11 as well as a test of delayed word recall and a number cancellation or maze task [47]
MMSE	The mini-mental state examination rates various cognitive domains, including memory, attention and language. Scores for MMSE range from 0 to 30; lower scores indicate greater cognitive dysfunction.[48]
MOCA	The Montreal cognitive assessment comprises 12 individual tasks (grouped into cognitive domains, including visuospatial and executive functioning, attention, language, abstraction, naming, delayed memory recall and orientation), which are mostly binary, and are assessed and summed with a 6-item orientation screening and an educational correction to determine a total score reflecting global cognitive functioning.[49]
FAQ	The Functional Activities Questionnaire evaluates the instrumental activities of daily living (IADLs), such as preparing meals and managing personal finances. The sum scores range in the 0-30 interval and the cut-point equal to 9 (dependent on 3 or more activities) is recommended to denote potential cognitive impairment.[50]
RAVLT	The Rey auditory verbal learning test involves five presentations of a 15-word list (List A), each followed by an attempted recall. This is followed by a second 15-word interference list (List B), followed by a recall of List A. It rates different aspects of episodic memory such as the learning rate (RAVLT learning and RAVLT immediate) and delayed recall (RAVLT percent forgetting) [51]
Ecog	The Everyday Cognition scale is an informant-rated questionnaire that includes one global factor and six domain-specific factors. The psychometric properties in the ECog scale address everyday function and cognition mild impairments reported from both participant (EcogPt) and study partner (ECogSP) [52]

## Methods

**Fig. 1:**
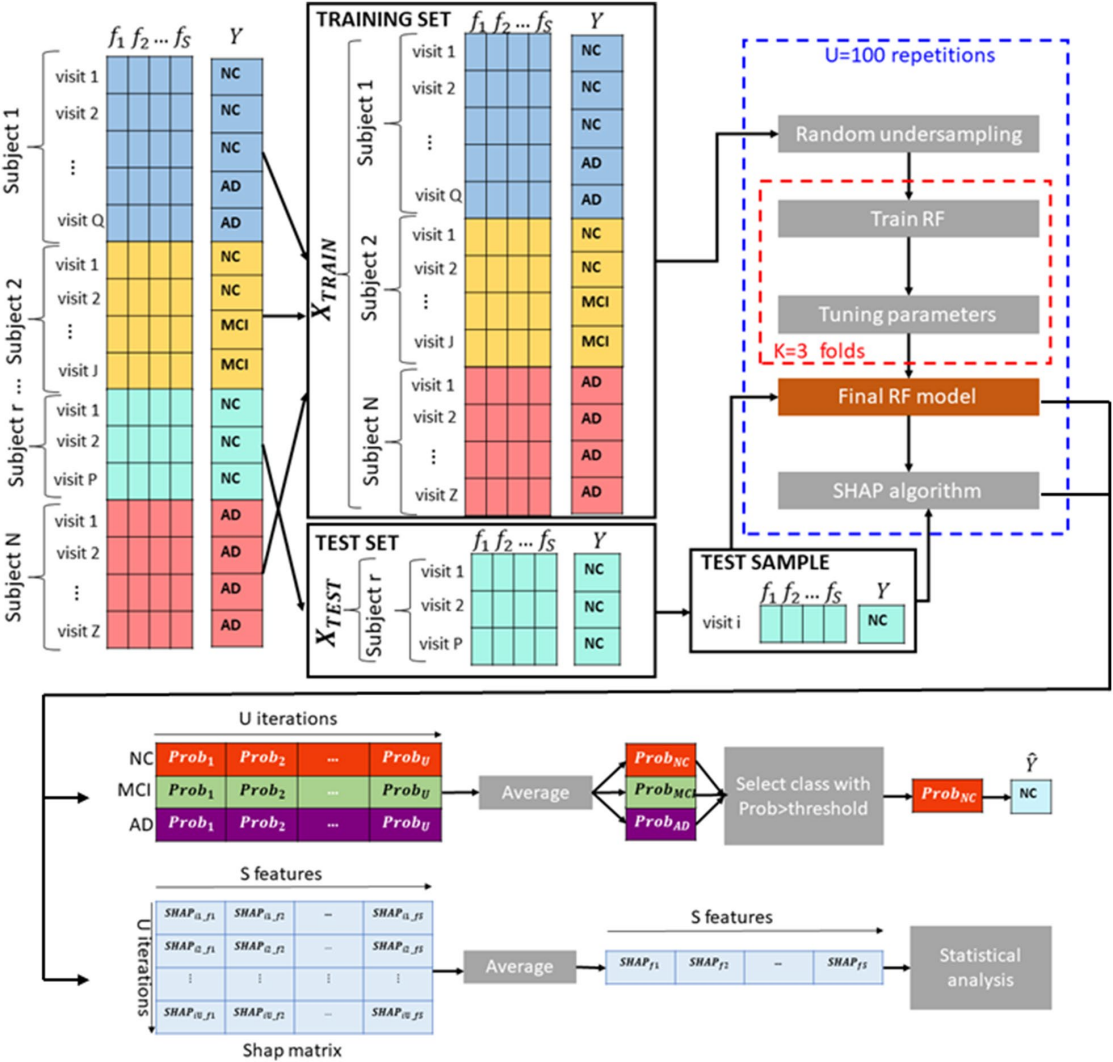
Workflow of the proposed analysis. The clinical and neuropsychological indexes (i.e., *S* features) are used to train a Random Forest (RF) classifier and predict the diagnosis of each subject at each visit with a leave-one-subject-out cross-validation strategy; for each cross-validation round the training set was randomly under-sampled $U = 100$ times by selecting a fixed amount of $N_{TRAIN}=500$ samples for each diagnostic category to handle class imbalance; the SHAP algorithm was used to explain the predictions of RF models for each sample; different statistical analyses were performed by using both probability scores resulting from RF and SHAP values to: (i) relate the performance of RF to the variability of the SHAP scores, (ii) analyze the variability of the SHAP scores between diagnostic categories, (iii) examine the longitudinal variability of the SHAP scores

Figure 1 shows the proposed workflow to both classify and quantify the cognitive variability in the selected cohort. The proposed approach comprises three main steps:

- The clinical and neuropsychological indexes are used to train a Random Forest (RF) classifier and predict the diagnosis of each subject at each visit (i.e., NC, MCI or AD);
- The SHAP algorithm is applied to explain the decisions made by RF for each sample. The output of SHAP is a vector of feature importance for each subject at a specific visit;
- Several statistical analyses are carried out within the framework of both a non-retrospective and a retrospective study. As part of the non-retrospective analysis, statistical comparisons were performed to: (i) relate the classification probabilities associated with the diagnostic category (as it was established by RF) for each example to the explanations associated with that decision; (ii) examine the similarity of SHAP indexes between groups of samples (both correctly and incorrectly classified); (iii) identify potential subgroups in the diagnostic groups provided by the RF classifier. Regarding the retrospective investigation, a statistical analysis was performed to compare the similarity of SHAP values between the first and the last visit of each participant in order to identify the cognitive index that best reflects the potential conversion of subjects from one diagnostic category to another.

Each step is detailed in the following sections.

### Predictive models

In this work, the RF algorithm was selected to predict the diagnostic label of each sample from a set of $S=10$ clinical and neuropsychological indexes. Given the dataset $D=\{X_i,y_i\}_{i=1}^N$, where $X_i$ is the vector of *S* features and $y_i$ is the target label for sample *i*, RF is an ensemble learner of decision tree base learners that individually predict the target response to output both the final prediction probability score and the target class of each sample by using a majority-voting mechanism [53]. Each decision tree is trained on bagged data (sampling with replacement) using a random subset of *mtry* candidate predictors. Random forest is one of the most widely used machine learning algorithms for the detection and classification of AD and MCI as it overcomes some of the disadvantages of decision trees such as multicollinearity and overfitting problems [54, 55].

In order to obtain clinically unbiased results, we adopted a leave-one-subject-out cross-validation strategy. According to this validation scheme, the dataset is split into as many sets as the number of subjects: one subject is randomly selected for testing while the others are used to train the model, and the procedure is repeated until all the subjects have been used as test [56, 57]. In our case, each subject has multiple samples that consist of a set of clinical and neuropsychological indexes measured at multiple visits, and all the samples belonging to the same subject are used within each test round.

Moreover, since in general, the ML algorithms can be sensitive to changes in the training set, returning unstable performance, for each cross-validation round we randomly under-sampled the training set $U = 100$ times by selecting a fixed amount of $N_{TRAIN}=500$ samples for each diagnostic category from the training set. This step can also handle class imbalance. A RF model was trained within each cross-validation round based on the grid search and nested k-fold stratified cross-validation (CV), with $k=3$. The entire training process has two loops: an inner loop for hyperparameter tuning (number of trees and *mtry*), and an outer loop for evaluating the trained model with the selected parameters on the unseen fold. This nested CV strategy has been adopted to avoid the use of the same data for parameter tuning and model evaluation and therefore to avoid overfitting. The tuned model was tested on each sample of the test subject to predict the diagnostic class, producing $U = 100$ probability scores for each class for each independent sample. Finally, each sample is assigned a label after averaging the $U = 100$ probability values for the three classes and selecting the class with the greatest probability score. This step was performed in order to maximize the generalization of the predictive models and obtain a highly stable final decision for each sample, possibly independent of the training set variability. We used the h2o.randomForest function implemented in the h2o (v.3.34.0.1) R package.

The performance of the models for each class *j* was evaluated by using the following metrics:

- Accuracy:
  1 $$\begin{aligned} \text {ACC}_{j}= \frac{\text {TP}_j+\text {TN}_j}{\text {TP}_j+\text {FP}_j+\text {TN}_j+\text {FN}_j} \end{aligned}$$
- Sensitivity:
  2 $$\begin{aligned} \text {SENS}_{j}= \frac{\text {TP}_j}{\text {TP}_j+\text {FN}_j} \end{aligned}$$
- Specificity:
  3 $$\begin{aligned} \text {SPEC}_{j}= \frac{\text {TP}_j+\text {TN}_j}{\text {TP}_j+\text {FP}_j+\text {TN}_j+\text {FN}_j} \end{aligned}$$
- Precision:
  4 $$\begin{aligned} \text {PREC}_{j}= \frac{\text {TP}_j}{\text {TP}_j+\text {FP}_j} \end{aligned}$$

Additionally, we reported the receiver operating characteristic (ROC) curves and the area under the ROCs (AUROCs) of one-versus-rest (OVR) decisions [58].

### Explainable machine learning

We selected the SHAP algorithm to explain the predictions of RF models for each sample. Indeed, SHAP is a local model-agnostic post-hoc explainer algorithm based on the Shapley value concept from game theory [59, 60]. It employs the output of a classifier, regardless of the specific model, and it learns an interpretable linear model at the local decision level, allowing one to explore the contributions of individual feature values on each prediction for a given test sample.

Let *D* be a dataset of samples, $D = [(\mathbf {x_1}, y_1), ( \mathbf {x_2}, y_2), ..., (\mathbf {x_N}, y_N)]$, where $\mathbf {x_i}$ represents the feature vector for the sample *i* and $y_i$ the corresponding label. Let *f* be a classifier and $f_{x_i}$ the prediction for the test instance *i* which corresponds to the predicted label.

The goal is to explain the contribution of each feature *j* among the *S* features as the average marginal contribution of the feature value across all possible coalitions, i.e., all possible sets of feature values with and without the feature *j*. In particular, a coalition, *F*, is defined to be a subset of *S* ($F \subseteq S$). If we denote with $f_{x_i}(F)$ the prediction for $f_{x_i}$ given the subset *F*, the following equation represents the marginal contribution of adding the *j*-th feature value to *F*:

5 $$\begin{aligned}{}[f_{x_i}(F \cup {j})-f_{x_i}(F)] \end{aligned}$$

To compute the exact Shapley value, all possible subsets of feature values excluding the j-th feature value $F\subseteq S - \{j\}$ have to be considered, hence:

6 $$\begin{aligned} \sum _{F\subseteq S - \{j\}} \frac{|F|!(|S|-|F|-1)!}{|S|!} [f_{x_i}(F \cup {j})-f_{x_i}(F)] , \end{aligned}$$

where |*F*|! represents the number of permutations of feature values positioned before the j-th feature, $(|S|-|F|-1)!$ represents the number of permutations of feature values that appear after the j-th feature value and |*S*|! is the total number of permutations [59].

The Shapley values are defined according to principles from cooperative game theory so that the resulting explanations satisfy some properties such as local accuracy, missingness, and consistency [59]. It is worth noting that, for a large number of variables *S*, the exact computation of the Shapley values could result not be feasible. Hence, different implementations of computations of Shapley values have been proposed, such as the Monte Carlo estimator [61] and TreeSHAP for tree-based models [59]. In this work, we adopted the DALEX R package to compute the SHAP scores for each test instance [62].

The absolute value of each SHAP score expresses how much each feature contributes to the final prediction [63]. It is important to note that local XAI methods considerably differ from feature selection techniques. Indeed, feature selection methods aim to determine the importance of each feature on a performance metric by using the training set. Thus, a single final feature importance vector is obtained, whereas SHAP returns one feature importance vector for each test instance.

### Statistical analysis of XAI scores

#### Non-retrospective analysis

In the non-retrospective analysis, we used all the samples from each subject to compare the distributions of SHAP values between the different clinical categories. Specifically, we divided the samples into seven classes according to the true and the predicted final label, i.e., (a) NC-NC (NC subjects correctly classified as NC), (b) NC-MCI (NC subjects incorrectly classified as MCI), (c) MCI-NC (MCI subjects incorrectly classified as NC), (d) MCI-MCI (MCI subjects correctly classified as MCI), (e) MCI-AD (MCI subjects incorrectly classified as AD), (f) AD-MCI (AD subjects incorrectly classified as MCI) and (g) AD-AD (AD subjects correctly classified as AD). The classes NC-AD and AD-NC were not considered due to the small number of subjects belonging to them.

We then assessed the similarity between each couple of samples within each class by computing the cosine distance between their SHAP vectors. A similarity network for each class has been defined for each class by including all the similarity scores between the samples within the class. Significant differences between the similarity networks across the classes were evaluated based on post-hoc comparisons ($p < 0.05$), following one-way analysis of variance (ANOVA) ($p < 0.05$).

Moreover, the stability-based k-medoid criterion proposed by [64] was applied to each similarity network to find the best partition into clusters. This criterion assesses the clusterwise stability of a dataset by resampling it several times with different methods such as bootstrapping or subsampling and by identifying the most stable clusters across the iterations. An important advantage of this method relies on the automatic assessment of the number of clusters through the stability criterion. We used the clusterboot function implemented in the fpc (v.2.2-9) R package, setting the bootstrap method and the number of clusters from 1 to 20. For each original feature, we compared the distributions of the resulting clusters by using one-way analysis of variance (ANOVA) or the Kruskal–Wallis test significant at $p < 0.05$ depending on the number of clusters. The main objective of this step was to investigate the heterogeneity of the categories provided by the classifier by detecting possible subgroups in each class and to explore the causes of the heterogeneity of the SHAP values as a function of the original feature values.

#### Retrospective analysis

In the retrospective analysis, we only analysed subjects with a number of visits $\ge 3$. For each subject, we calculated the cosine distance between the SHAP vectors of the first visit and the last visit. We then considered the following classes of subjects: (a) stable NC (diagnosed NC at baseline who remained NC at the last visit), (b) stable MCI (diagnosed as MCI at baseline who remained MCI), (c) stable AD (diagnosed AD at baseline who remained AD), (d) NC conv MCI (diagnosed NC at baseline visit who progressed to MCI), (e) NC conv AD (diagnosed NC at baseline visit who progressed to AD), (f) MCI conv AD (diagnosed MCI at baseline visit who progressed to AD). Significant differences of the cosine distance between the first and last visit across classes were evaluated based on post-hoc comparisons ($p < 0.05$), following a one-way analysis of variance with demographic variables as covariates (ANCOVA) ($p < 0.05$).

A non-parametric permutation test was also performed to identify the indexes that are significantly associated with conversion from one diagnostic category to another. Specifically, we assessed the statistical significance of the above-chance average cosine distance between the SHAP scores of the first and last visit for each class by using 1000 permutations.

## Results

### Performance of the predictive models

**Fig. 2:**
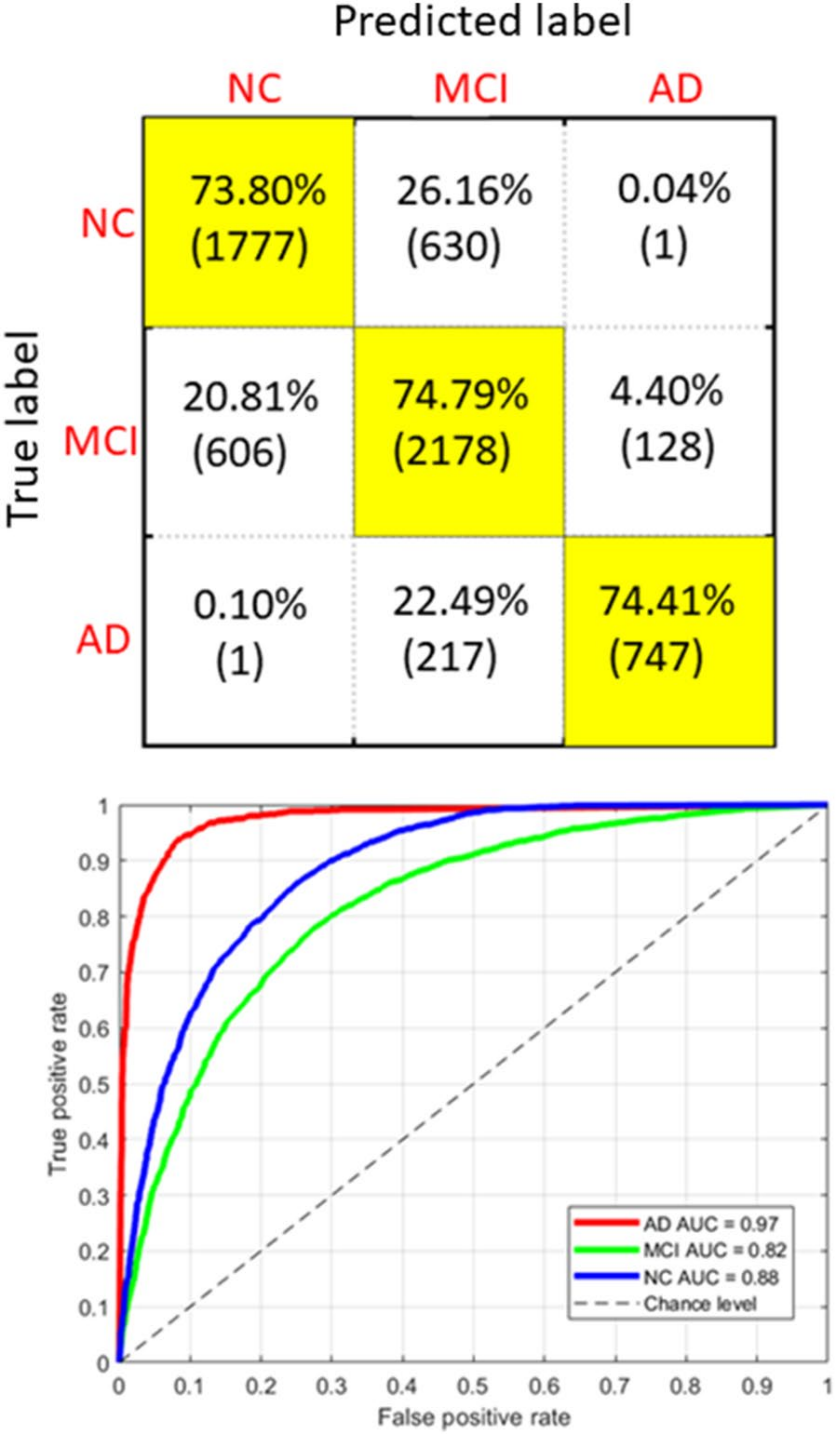
Performance of RF models: confusion matrix (top) and ROC curves (bottom)

The performance of the RF models is shown in Table 3. Figure 2 shows the confusion matrix for the three-classes classification problem over the entire dataset and the AUC curves for each class obtained with a one-versus-rest (OVR) strategy. As expected, the best performance is obtained for the AD class, which is the most distinguishable from the other two classes (AUC = 0.97). However, a large percentage of the samples from this class ($22.5\%$) are systematically classified as belonging to the MCI class, showing a high overlap of the neuropsychological scores with MCI subjects. Similarly, $20.8\%$ of the samples belonging to the MCI class are systematically classified as NC and $26.2\%$ of the NC samples are incorrectly classified as MCI. These outcomes show that, from the cognitive perspective, the three diagnostic categories are not markedly separate, but substantial overlaps can be observed between certain classes, prompting further analysis of their explainability.

**Table 3:** Performance metrics of the RF models

Model	Accuracy	Specificity	Sensitivity	Precision	AUC
NC	$74 \pm 4\%$	$84 \pm 3\%$	$73 \pm 4\%$	$70 \pm 8\%$	0.88
MCI	$75 \pm 3\%$	$75 \pm 4\%$	$75 \pm 4\%$	$70 \pm 7\%$	0.82
AD	$72 \pm 5\%$	$97 \pm 2\%$	$72 \pm 3\%$	$80 \pm 5\%$	0.97
Global	$75\%$	$85\%$	$74\%$	$73\%$	0.89

### Statistical analysis of XAI scores

#### Non-retrospective analysis

**Fig. 3:**
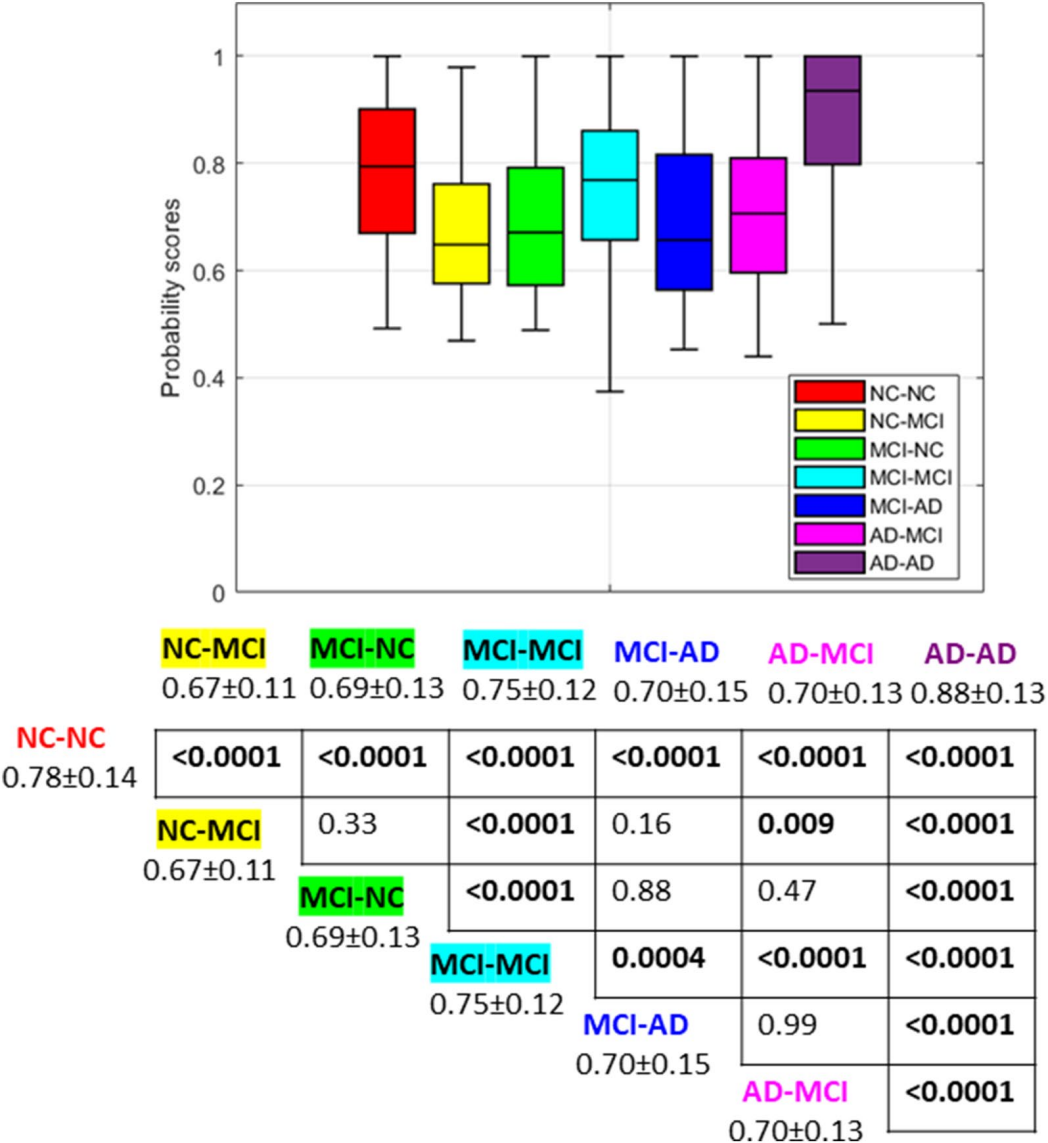
Comparisons of probability scores across diagnostic classes: boxplots (top) and table with p values resulting from post-hoc analysis with average and standard deviations for each class (bottom)

**Fig. 4:**
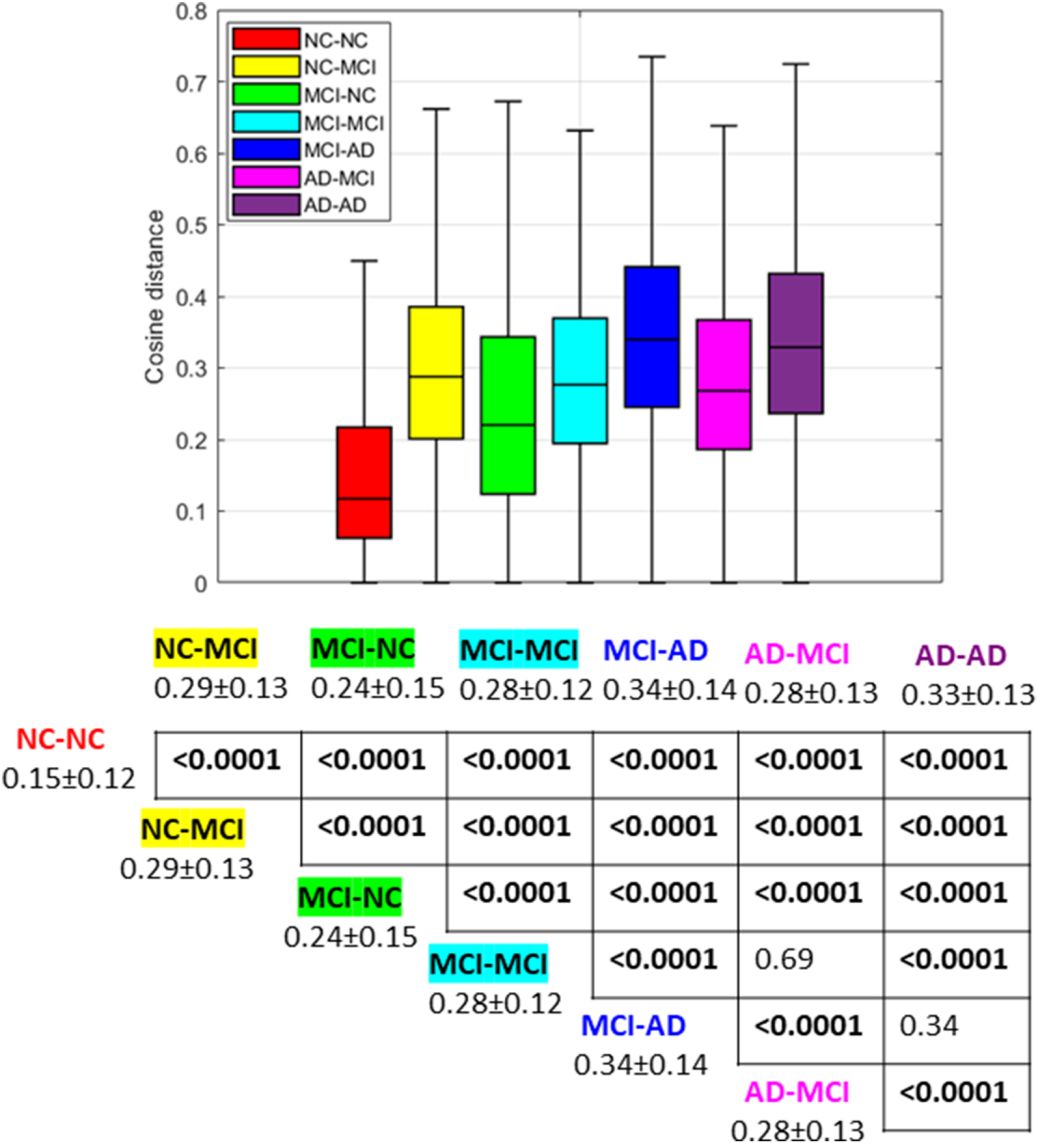
Comparisons of cosine distances between the SHAP vectors of each possible pair of subjects belonging to each class: boxplots (top) and table with p values resulting from post-hoc analysis with average and standard deviations for each class (bottom)

After obtaining a single vector of SHAP values for each sample, we constructed a similarity matrix for each diagnostic category, both correctly classified and systematically misclassified, by calculating the cosine distance between the SHAP vectors of each possible pair of subjects belonging to each category. Figures 3 and 4 show the boxplots of the distributions of the decision probability scores of the RF algorithm and of the cosine distance values for the categories.

Between-group comparisons and post-hoc analysis (bottom parts of Figs. 3 and 4) revealed that the probability scores of the RF models for the correctly classified samples are significantly different from the decision probability values of the misclassified categories, and in particular, the RF models show the highest probability scores for the AD-AD class. However, the distribution of the cosine distances between the SHAP vectors of the samples of the class AD-AD is not statistically different from the distribution of the MCI-AD category, showing heterogeneous importance of clinical and cognitive scores similar to that of the samples belonging to the MCI-AD group.

**Fig. 5:**
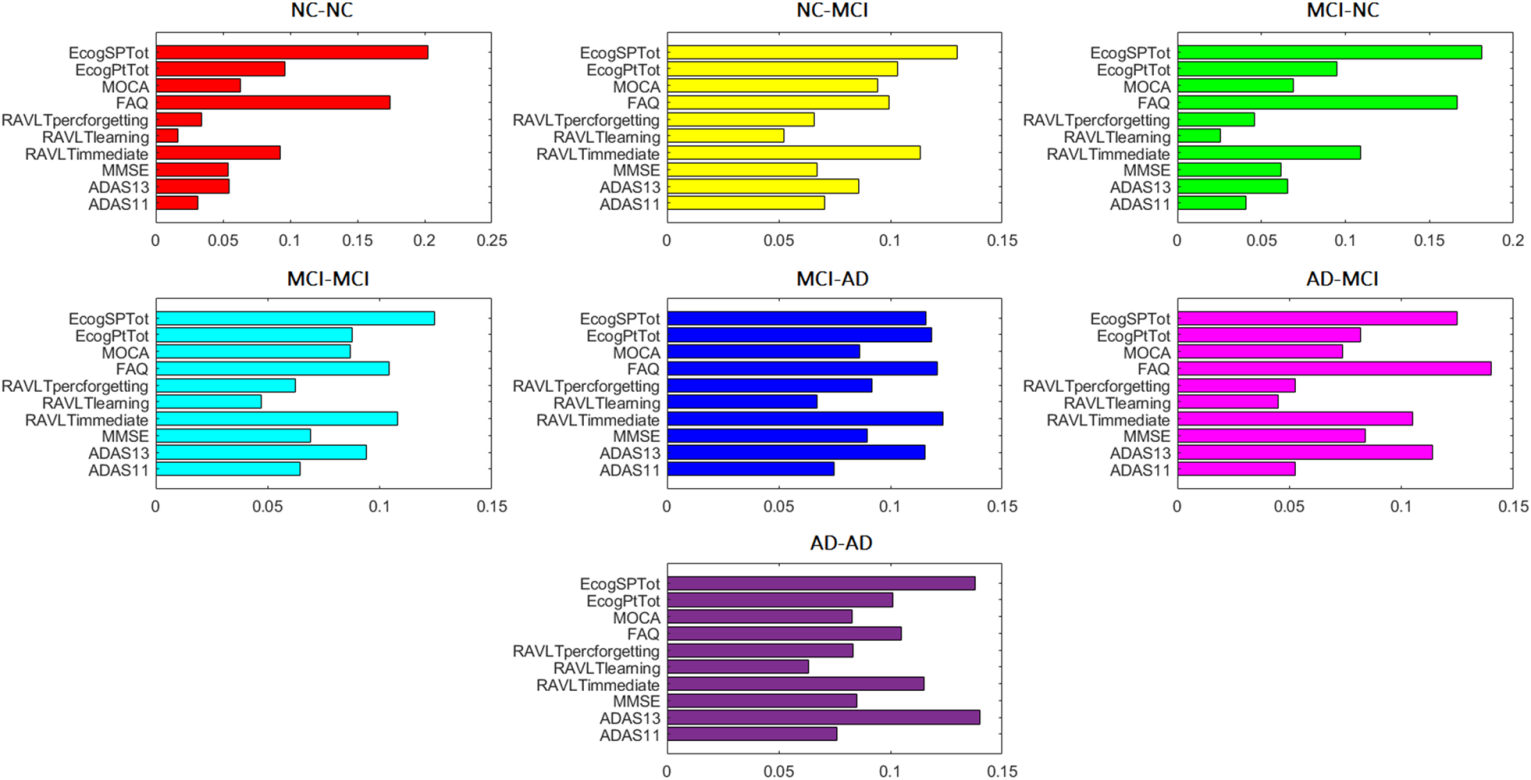
Variable importance plot showing the average SHAP values for each index within each class

In Fig. 5 we also reported the variable importance plot for each class, showing the SHAP values for each index averaged over the samples within that class. This plot allows an immediate comparison of the importance ranking of the clinical and cognitive variables for each class obtained from the classifier. Very similar patterns of importance ranking can be noted between NC-NC and MCI-NC; NC-MCI, MCI-MCI, and AD-MCI; MCI-AD, and AD-AD.

By applying the stability algorithm to each similarity network, we identified two clusters for the categories. For each original feature, we compared the distributions of the resulting clusters by using Student’s t-test, significant at $p < 0.05$. Additional file 1: Table S1 shows that the two clusters identified in the similarity network of NC-MCI samples differ in almost all clinical and cognitive indexes, highlighting that this classification category is actually composed of two groups with very different clinical and cognitive profiles. For the other two categories, significant differences occur between the two subgroups for a limited subset of cognitive indexes which overlap almost completely (such as ADAS11, MMSE and FAQ).

#### Retrospective analysis

**Fig. 6:**
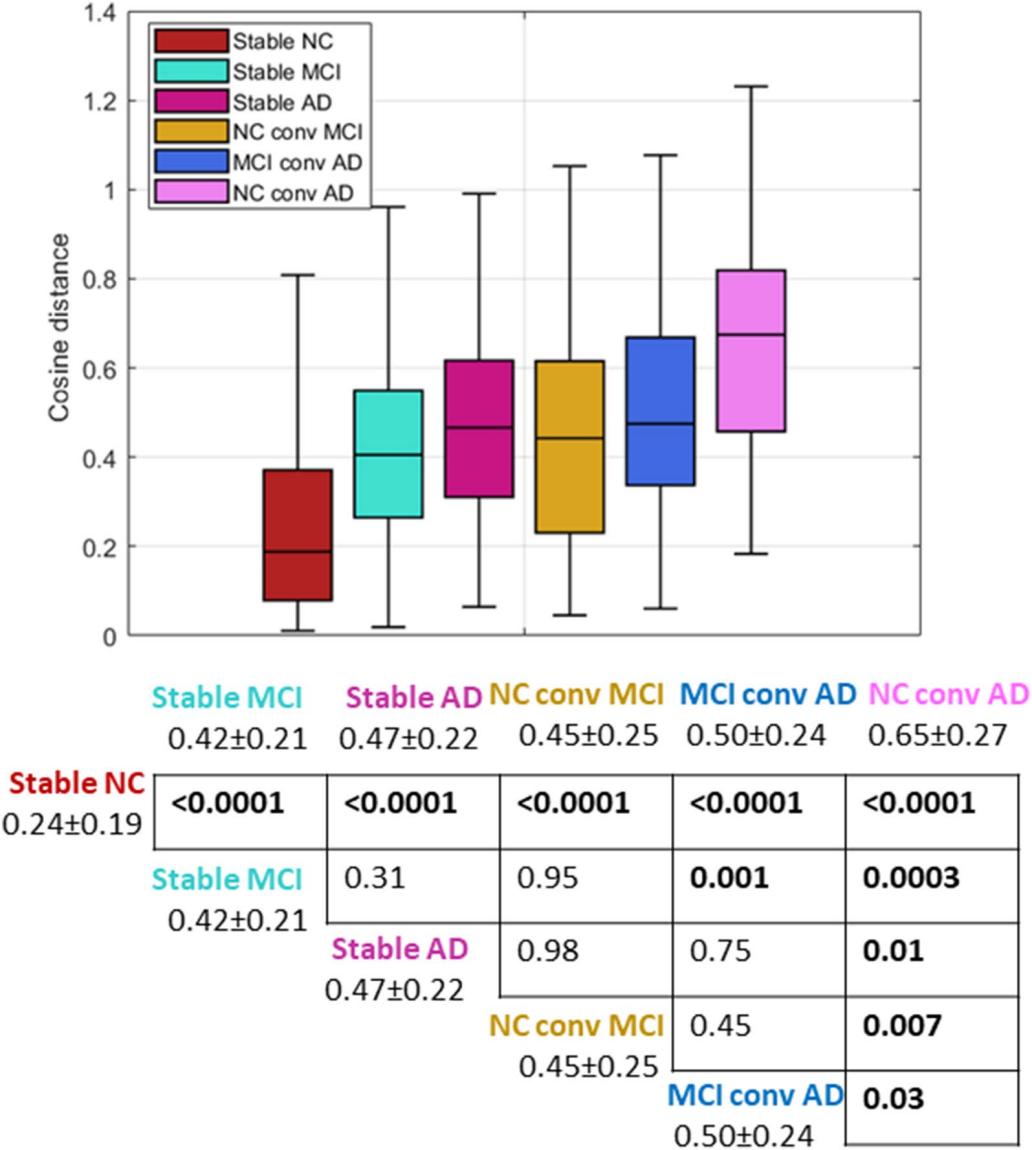
Comparisons of cosine distances between the SHAP vectors of the first and last visits of the subjects for the diagnostic longitudinal categories: boxplots (top) and table with p values resulting from post-hoc analysis with average and standard deviations of probability scores for each category (bottom). *p* values in bold are statistically significant

Figure 6 shows the boxplots of the distributions of the cosine distance between the SHAP vectors of the first and last visits of the subjects for the diagnostic longitudinal categories. Between-group comparisons and post-hoc analysis in the bottom part of Fig. 6 revealed that the stable NC and NC converter to AD were significantly different from other groups. In particular, the stable NC subjects show the lowest variability between the SHAP values of the first and last visits, meaning more similar contributions of the clinical and neuropsychological scores to the RF decisions. In contrast, the NC subjects converter to AD show the highest distance between the score contributions of the first and last visit. In addition, the cosine similarity values of the stable MCI subjects were significantly lower than those of the MCI converter to AD subjects. These findings highlight that SHAP scores reflect the extent of conversion and therefore could be appropriately used to track longitudinal changes in the contributions of the distinct cognitive domains to the patient’s condition.

**Fig. 7:**
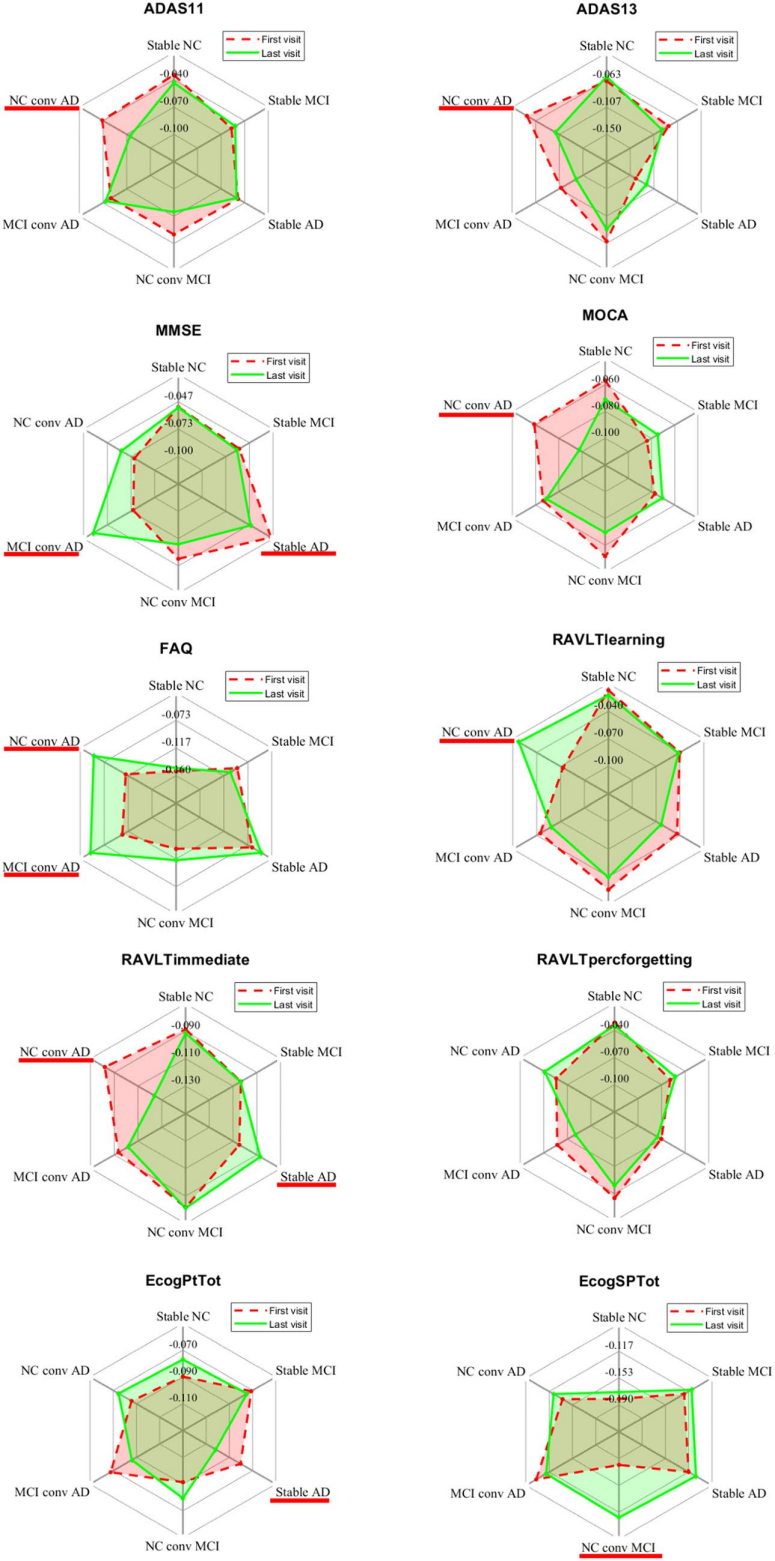
Radar plots reporting the average SHAP values for the first and last visit for each category and each cognitive index

Figure 7 shows the radar plots of the average SHAP values for the first and last visit for each category and each cognitive index.

The indexes with significant longitudinal changes in SHAP values are underlined in red ($p<0.05$ resulting from the permutation test). The highest number of significant longitudinal changes in the SHAP values was found for the NC subjects converting to AD, while no significant longitudinal changes were detected for stable categories.

## Discussion

In this work, we explored the predictive power of different neuropsychological measures to discriminate between different degrees of cognitive impairment. Accordingly, we set up a three-class classification problem (i.e., NC/MCI/AD) following the diagnostic categories assigned to patients at each visit based on the ADNI consortium criteria. Although a reduced subset of features was selected in this study for the benefit of larger sample size, the RF model achieves state-of-the-art performances [65, 66]. In particular, our results showed that with 10 features, we achieved an overall accuracy of $70\%$ and AUC=0.89. In accordance with other works, we reached the maximum specificity and AUC performance value for the AD class, indicating that the selected cognitive scores are most effective in detecting Alzheimer’s disease that corresponds to the latter part of the neurodegeneration spectrum. On the other hand, our findings also indicate that a discrete classification approach may fail to capture information on the cognitive impairment across the spectrum and some subjects may be repeatedly misclassified due to intermediate cognitive conditions between two diagnostic categories [67]. In order to exploit both clinical information and investigate the presence of intermediate classes between the discrete diagnostic categories, we also considered the misclassification labels, i.e., CN-MCI, MCI-CN, MCI-AD, and AD-MCI. Indeed, the developed framework consists of retraining $U=100$ models that are tested on each independent subject, and consequently, if a sample is misclassified a large number of times, it is reasonably considered as “systematically misclassified” according to a robust quantitative criterion.

We investigated the heterogeneity of the impact of different cognitive and neuropsychological indexes on the prediction of the clinical outcome using the local XAI SHAP method. The main objective of this step was to understand if the whole XAI vector of feature importance can be exploited as a new derived cognitive marker across the cognitive spectrum instead of using a set of original features. Indeed, this method would allow a more conscious use of artificial intelligence methods in clinical practice. As a first step, we, therefore, examined the intra-group similarity of XAI vectors through the cosine distance measure. The statistical analysis carried out on the different groups of subjects showed that the healthy subjects (i.e., those belonging to the NC-NC class) presented the lowest intra-group variability of the XAI vectors as reported in Fig. 4. This finding highlights that the SHAP vectors exhibit high sensitivity by showing a marked separation of the cosine distance distributions of the other classes. On the other hand, the AD-AD and MCI-AD classes were found to be the most heterogeneous categories since their intra-group distribution of cosine distance values was significantly higher than those of the other diagnostic categories. This result indicates that cognitive decline in the latter part of the neurodegeneration spectrum is more varied in accordance with the observed larger degree of cognitive variability among individuals with AD dementia compared to individuals in predementia stages [68]. Interestingly, the intra-class variability of the SHAP vectors does not have a trivial correspondence with the classifier’s probability scores: although the NC-NC and AD-AD classes have the highest probability values, they show very different cosine distance distributions. We further examined the intra-class variability of the SHAP vectors through clustering analysis. The results show that only some inter-diagnostic groups such as MCI-AD, AD-MCI, and MCI-NC are further separable into two other subgroups. For each original feature, the distributions of the resulting clusters were compared by using Student’s *t*-test, significant at $p <0.05$. As reported in Additional file 1: Table S1, we found that despite the two clusters within each of the two classes MCI-AD and AD-MCI differ in quite the same indexes that refer to memory domain values within general cognitive assessment (e.g., ADAS11 and MMSE), they exhibit different average values, showing a marked further categorization of the classes.

We showed the variable importance plots in Fig. 5 for the global interpretability of the indexes. It can be observed that both the FAQ and EcogSPTot indices prevail for NC-NC and MCI-NC. Moreover, for the MCI categories (correctly classified and misclassified), the importance of all the other variables increases, and for the categories classified as AD (correctly classified and misclassified), the index ADAS13 also becomes particularly relevant, thus indicating that this variable is particularly important for detecting the last spectrum of neurodegeneration.

A retrospective analysis of the SHAP scores was performed to investigate the longitudinal variation of the SHAP vectors for different diagnostic categories. In particular, we compared the similarity of SHAP values between the first and the last visit of each participant for both converting and stable clinical classes. In this case, stable NC subjects show the least variability (see Fig. 6), indicating that cognitive ageing trajectories are markedly less variable in subjects without neurodegenerative diseases. On the contrary, for stable MCI and AD subjects, we found similar longitudinal variability of the SHAP vectors, showing more varied cognitive ageing trajectories. Interestingly, the highest longitudinal variability of SHAP vectors is observed for the class of NC converter to AD, showing that the cosine distance between the explainability values could effectively reflect the degree of conversion. By using the non-parametric statistical tests, we obtained the list of indexes with SHAP scores significantly associated with the longitudinal cognitive variability: as shown in Fig. 7, no index has significant variation in stable subjects of any diagnostic category, with the exception of AD subjects, for which significant longitudinal variation of the SHAP scores is observed in the indexes MMSE, RAVLTimmediate and EcogPtTot. For the NC-to-MCI converters, we found that EcogSPTtot was the only cognitive index whose SHAP values varied significantly longitudinally, confirming that the study partner report can discriminate between, and predict progression from, cognitively normal status to mild cognitive impairment [69, 70]. Finally, it is noteworthy that the highest number of cognitive indexes whose impact is significantly different between the first and the last visit was found in NC subjects with rapid conversion to AD, highlighting that these subjects have evident impairment in almost all the cognitive and functional domains.

## Limitations and future perspective

In this work, SHAP achieves to explain the internals of a RF classifier trained on cognitive and clinical information, thus showing a possible link between diagnosis and patterns of feature relevancy. However, neuropsychological assessment represents a preliminary step for the clinical diagnosis of AD and using other exams and neuroimaging data might largely improve the diagnostic results and add validity to the interpretation of predictions. Several works have shown that the accuracy resulting from the three-class (AD vs. MCI vs. HC) classification task can be considerably increased by using MEG data, PET, SPECT and MRI imaging modalities properly integrated with convolutional architectures and deep learning models [71–73]. In future developments, we will examine the XAI scores resulting from the classification with neuroimaging data to investigate the patterns of feature relevancy for the biological variables. In addition, we will perform a correlation analysis between the importance of clinical and cognitive variables and those derived from the imaging modalities to provide deeper insights into the connection between the cognitive status and the biological features throughout the neurodegenerative process.

## Conclusions

In this work, we provided a ML framework to explore the contribution of cognitive and clinical measures for the automatic classification of mild cognitive impairment and Alzheimer’s disease. We demonstrated that explainability must be related to ML efficiency in order to obtain reliable characterisations of patients’ cohorts. This is of paramount importance for XAI to provide valuable insights about the disease progression. Hence, we developed a robust ML algorithm with a XAI module to shed more light on the impact of cognitive and clinical indexes on the diagnostic category assigned to patients at each visit. A statistical analysis of the XAI vectors revealed that diagnostic categories in the last part of the neurodegenerative spectrum have the greatest variability, which can be explained by the existence of different subcategories within those considered in the study. Moreover, a retrospective analysis clearly outlined that the impact of cognitive and clinical indexes does not vary substantially for stable subjects, regardless of the starting diagnostic category, and that for specific converting categories of subjects, it might be more appropriate to consider only the limited set of indexes that show a significant longitudinal change in their impact on the final prediction of the diagnosis. Our findings allow us to state that the SHAP values can effectively characterise the impact of each index on the cognitive status of patients and quantify the variation of such impact over time, keeping track of longitudinal changes and providing continuous information about progression to AD, in line with the current 2018 NIA-AA research framework that has transitioned to defining AD as a continuum.

## Supplementary Information

**Additional file 1: Table S1.** Mean and standard deviation values for the two clusters identified in the similarity network for each group. Significant differences resulting from Student's t-test are shaded in gray.

## Data Availability

The dataset that supports the findings of this study is publicly available on databases cited in the bibliography.

## Abbreviations

AD: Alzheimer’s disease
ADAS11: Alzheimer’s Disease Assessment-Cognitive 11-item scale
ADAS13: Alzheimer’s Disease Assessment-Cognitive 13-item scale
ADNI: Alzheimer’s Disease Neuroimaging Initiative
MCI: Mild cognitive impairment
ML: Machine Learning
NC: Normal control
ECog: Everyday cognition
ECogPtTotal: ECog participant total
ECogSPTotal: ECog study partner total
FAQ: Functional Assessment Questionnaire
GAN: Generative Adversarial Network
MMSE: Mini-Mental State Examination
MOCA: Montreal Cognitive Assessment
MRI: Magnetic resonance imaging
PET: Positron emission tomography
RAVLT: Rey Auditory Verbal Learning Test
RAVLTimmediate: Rey Auditory Verbal Learning Test Immediate
RAVLTlearning: Rey Auditory Verbal Learning Test Learning
RAVLTpercforgetting: Rey Auditory Verbal Learning Test percent forgetting
RF: Random forest
ROC: Receiver operating characteristic
SHAP: SHapley Additive exPlanations
XAI: eXplainable Artificial Intelligence
