# Supplementary material for: A robust framework to investigate the reliability and stability of explainable artificial intelligence markers of Mild Cognitive Impairment and Alzheimer’s Disease
Source: Brain Inform. 2022 Jul 26;9(1):17. doi: 10.1186/s40708-022-00165-5 (PMC9325942; doi:10.1186/s40708-022-00165-5)
Supplement: Supplementary file 1 — Additional file 1: Table S1. Mean and standard deviation values for the two clusters identified in the similarity network for each group. Significant differences resulting from Student's t-test are shaded in gray. [file 40708_2022_165_MOESM1_ESM.pdf]

Additional file

A robust framework to investigate the reliability and stability ofexplainable artificial intelligence markers of Mild Cognitive Impairment and Alzheimer’ s Disease

Angela Lombardi Domenico Diacono Nicola Amoroso Przemyslaw Biecek Alfonso Monaco

Loredana Bellantuono Ester Pantaleo Giancarlo Logroscino Roberto De Blasi Sabina Tangaro Roberto Bellotti

Supplementary Table 1: Mean and standard deviation values for the two clusters identified in the similarity network for each group. Significant differences resulting fromStudent’s t-test are shaded in gray.

Group	ADAS11	ADAS13	MMSE	RAVLTimm	RAVLTlearn	RAVLTpercforg	FAQ	MOCA	EcogPtTot	EcogSPTot
AD-MCI	$C_1 = 14.5 \pm 4.8$	22.7 ± 6.5	25.4 ± 2.2	28.8 ± 8.3	2.6 ± 2	79.7 ± 27	7.1 ± 4.5	20.9 ± 3.3	2 ± 0.7	2.3 ± 0.6
	$C_2 = 13.2 \pm 5.2$	21.1 ± 6.7	26.4 ± 2.3	27.2 ± 8.7	3.2 ± 2.3	77.9 ± 30	9.1 ± 5.7	21.5 ± 3.7	1.9 ± 0.5	2.5 ± 0.7
MCI-AD	$C_1 = 19.2 \pm 5.2$	29 ± 6.2	23.5 ± 2	21.6 ± 6.7	1.9 ± 1.7	93.2 ± 14.3	9.5 ± 5.9	18.6 ± 3.2	1.9 ± 0.6	2.7 ± 0.5
	$C_2 = 17.3 \pm 5.2$	27 ± 6.4	25 ± 2.7	25.9 ± 6.9	2.4 ± 2	89.7 ± 19.2	13.1 ± 5.3	19.6 ± 3.4	1.9 ± 0.6	2.7 ± 0.6
MCI-NC	$C_1 = 6.8 \pm 3.3$	10.5 ± 4.9	28.8 ± 1.2	43 ± 10.4	5.7 ± 2.4	38.2 ± 28.5	0.1 ± 0.4	25.1 ± 2.7	1.4 ± 0.3	1 ± 0.08
	$C_2 = 5.8 \pm 2.7$	8.4 ± 3.7	29 ± 1.2	50 ± 9.8	6.1 ± 2.2	29.7 ± 28.1	0.3 ± 0.7	26.5 ± 2.3	1.4 ± 0.3	1.3 ± 0.2
